# Acute Nephritis with Extreme Hæmaturia

**Published:** 1908-01-25

**Authors:** 


### Illustrative Cases.

### ACUTE NEPHRITIS WITH EXTREME HÆMATURIA.

Acute Bright's disease is often enough associated with hæmaturia; but the amount of blood lost in the urine in this way is seldom enough to give rise to pronounced anæmia of itself. The following patient suffered from such a marked degree of hæmaturia that for many weeks the urine looked like pure blood —a degree of hæmaturia that is not often seen apart from calculous disease, or villous or other tumour. The case is further of interest in two other respects, namely: First, that notwithstanding the extreme degree of nephritis that must have been present to produce so much and such prolonged renal hæmorrhage, complete recovery occurred for the time being. Secondly, that although the attack passed off, and the urine became entirely free from albumen and renal tube casts, there was, in all probability, a residuum of renal inflammation ready to light up again on the slightest provocation. We have elsewhere laid stress upon the fact that absence of albumen from the urine by no means necessarily implies that kidneys which have been the seat of acute nephritis have become entirely restored to health.

The patient was a man aged thirty-five, a porter by trade, who was much exposed to inclemencies of weather by reason of his occupation. He got wet through one day, and was unable to change his clothes promptly. A bad cold followed, and a week later he found that his face and arms were swollen, and in two or three days the swelling extended to the legs. He still tried to go on with his work, but intense headaches compelled him to go to hospital a fortnight after the beginning of his illness. He then exhibited the usual œdema of nephritis in his legs, scrotum, loins, abdominal wall, arms, and face.

The quantity of urine passed daily was greatly diminished; its specific gravity was 1,025; it had a dusky hue; on boiling it went nearly solid with coagulated albumen, and microscopically there were red corpuscles and renal tube casts of all kinds in abundance in the deposit.

Under the use of hot air baths, digitalis and potassium acetate, the urine increased in quantity and diminished in specific gravity; but from being merely dusky it soon became increasingly red until, twelve days after admission, it assumed the colour of blood, so highly charged was it with that fluid.

The dropsical symptoms showing no marked tendency to increase nor yet to subside, and the patient becoming decidedly blanched from loss of blood, the restraint of the hæmorrhage seemed to be the most urgent point of treatment if it could be accomplished. Accordingly, the use of various reputed styptics was tried. Acetate of lead was given first; this very soon produced a well-marked blue line upon the gums but no other effect. After a week's trial it was abandoned for the tincture of perchloride of iron, which was given alone in water, in doses of fifteen minims three times a day for four days; after this it was continued for three days in combination with tincture of digitalis and tincture of opium. These remedies seemed to have no effect whatever in restraining the hæmorrhage. The quantity of fluid passed from the bladder—blood and urine together—was now more abundant.

The hæmorrhage being as great as ever a month after admission, and the dropsy undiminished, it was decided to try tannin, and at the same time to administer a ferruginous wine. The patient was ordered three grains of tannin in an ounce and a half of decoction of uva ursi thrice daily, and he was allowed six ounces of the red wine. The bowels were kept open by occasional enemata, and by compound powder of jalap. Three days later the dose of tannin was increased to six grains; and again, three days later to ten grains. The hæmaturia was as bad as ever, the patient was quite blanched and had the look of one who had had a severe hæmorrhage, together with persistence of the renal dropsy.

Soon after the tannin was increased to ten grains three times daily the reports began to be more favourable. It is very doubtful, of course, whether the tannin really had anything to do with the improvement, for the latter may quite well have been due to spontaneous decrease in the acuteness of the nephritis. Nevertheless, the œdema now became less; and while there was no diminution in the quantity of the urine, its colour became a less deep red. A week later the dose of tannin was increased to fifteen grains. The hæmaturia steadily decreased until, eight weeks after the patient first came in, it was thought wise to stop the tannin and to give citrate of iron, in consequence of the man's extreme anæmia. The latter was now so great that, in addition to the usual blanched appearance of the skin and mucous membranes, there was a loud hæmic bruit over the pulmonary artery, and a bruit de diable over the veins in the neck; and the patient was greatly troubled with buzzing noises in his ears.

After another month's treatment of this kind, the hæmorrhage had ceased, the dropsy was quite gone, urine, of specific gravity 1,013, was secreted in normal quantities; and it contained a very slight quantity of albumen. He had gained so much in general health that he was allowed to leave the hospital after a sojourn in it of three months, during two of which he was passing blood in such large quantities that the urine looked like pure blood.

The subsequent history of the case is of some interest. He became an inmate of the hospital three times during the four subsequent years, for various minor complaints. Opportunity was thus afforded for examining the urine, and on each and all of these occasions it was free from albumen, blood and renal tube casts, and no evidence of disease of the kidneys could be obtained. Four years afterwards, that is to say eight years after the original attacks, he was again admitted for dropsy and alumbinous urine. Twelve years after his first attack he had another and similar attack.

In neither of the subsequent exacerbations of the nephritis was there hæmaturia in any way comparable with the severity of that of his original attack.

